# Exosomic microRNAs in the Tumor Microenvironment

**DOI:** 10.3389/fmed.2015.00047

**Published:** 2015-07-22

**Authors:** Paolo Neviani, Muller Fabbri

**Affiliations:** ^1^Children’s Center for Cancer and Blood Diseases, The Saban Research Institute, Children’s Hospital Los Angeles, Los Angeles, CA, USA; ^2^Department of Pediatrics, Norris Comprehensive Cancer Center, Keck School of Medicine, University of Southern California, Los Angeles, CA, USA; ^3^Department of Molecular Microbiology and Immunology, Norris Comprehensive Cancer Center, Keck School of Medicine, University of Southern California, Los Angeles, CA, USA

**Keywords:** microRNAs, exosomes, tumor microenvironment, toll-like receptors, inflammation, cancer

## Abstract

Dissecting the crosstalk between tumor cells and tumor microenvironment is quickly becoming the new frontier in cancer research. It is now widely accepted that cancer cells can exert a profound influence over their surroundings, by changing the microenvironment from a normal to a tumor-supportive state that allows for sustained tumor growth, invasion, and drug resistance. Extracellular vesicles, especially exosomes, are recognized as a new category of intercellular communicator, and they are emerging as of primary importance in controlling the interplay between the tumor and its environment. Exosomes derived from cancer cells or from cells of the tumor microenvironment allow for the horizontal transfer of information by virtue of their cargo, made of functional proteins and nucleic acids that are specifically sorted and loaded in exosomes during their biogenesis. In this review, we will discuss the current knowledge regarding the role invested by microRNAs, a family of short non-coding RNAs frequently deregulated in malignancies and present in exosomes, in shaping the microenvironment in a cancer-dependent manner.

## Introduction

While great advances have been made in the fight against cancer, it is still a leading cause of death in the twenty-first century, and a deeper understanding of the biology of the cancer cells and their surrounding is needed in order to develop novel therapeutic strategies. In the past three decades, researchers and clinicians have mostly focused on the identification of cancer-specific targets and the development of targeted therapies that could efficiently kill the cancer cells while sparing their normal counterpart, therefore reducing unwanted side effects. This global effort has resulted in the development of a number of promising and highly effective small molecules targeting cancer-specific alterations and/or altered signal transduction pathways that control the proliferation and survival of cancer cells (1). Moreover, a series of monoclonal antibodies have been developed, and FDA approved, to exploit the preferential and/or enhanced expression of antigens on the surface of malignant cells (e.g., ERBB2 in breast cancer, CD20 or CD52 in chronic lymphocytic leukemia) and to allow the specific modulation of survival pathways (when utilized as naked antibodies), or the targeted delivery of a toxic payload (when utilized as conjugated antibodies) (2). Although this approach has proven effective and promising in some cases, there are obvious drawbacks; first, because of the clonal evolution of malignant cells, cancers are characterized by extensive heterogeneity and a variety of subtypes that makes it difficult to identify unique targets and to eradicate the totality of tumor cells; second, it is important to remember that the growth of a tumor may also be influenced by its own surroundings and the host organism as a whole. Indeed, there is increasing evidence that the interaction between tumor cells and components [i.e., stromal cells, immune cells, and extracellular matrix (ECM)] of the surrounding tumor microenvironment may directly affect the growth and the drug resistance of the primary tumor and also control its evolution from early to late/metastatic stages.

## The Tumor Microenvironment

The tumor microenvironment is defined as the variety of normal cells, blood vessels, signaling molecules, and ECM that surround the tumor cells. The cellular components of the tumor microenvironment include endothelial cells, pericytes, fibroblasts, and immune cells (3). Importantly, one of the first mechanisms of interaction between the tumor and the microenvironment to receive considerable attention was the discovery that tumors can secrete factors (e.g., VEGF) that act on neighboring endothelial cells resulting in the formation of new blood vessels able to support the continuous growth and the metastatic potential of the tumor itself (3). Tumor-associated immune cells are also important components of the tumor microenvironment; in particular, tumor-associated macrophages (TAMs) have been reported to be frequently detected in close proximity of tumors or infiltrating its stroma (4). Interestingly, solid tumors can be stratified based on the level of TAM infiltration, and high levels of TAMs frequently correlate with enhanced metastatic potential, increased vascularization, and overall poor prognosis (4). Indeed, naturally occurring TAMs are generally skewed toward an M2-like activated phenotype (CD68+, CD163+), and display overall pro-tumorigenic and pro-angiogenic activities, suggesting that persistent inflammation at the tumor site may contribute to tumorigenesis (3, 5, 6). Other tumor microenvironment components, cancer associate fibroblasts (CAFs), mesenchymal stem/stromal cells (MSCs), cancer-associated adipocytes (CAAs), and the ECM, also play a role in supporting cancer cell growth and dissemination (7). In particular, CAFs and MSCs in the tumor microenvironment exist in a pro-inflammatory state and secrete inflammatory chemokines and growth factors (i.e., CCL2, CXCL8, CCL5, FG9) with known pro-tumorigenic functions (8, 9). CAAs and CAFs also contribute to structural remodeling of the ECM, through aberrant deposition of collagen, laminin, fibronectin, and secretion of matrix metallo-proteases (MMPs), hence supporting not only local tumor growth but also basal membrane invasion and cancer cell metastasis (7). Like TAMs, high infiltration of perpetually activated pro-inflammatory CAFs and CAAs has been linked to poor prognosis in several malignancies, such as non-small cell lung cancer (NSCLC) and breast cancer (7). Importantly, it is becoming evident that the cross-talk between cancer cells and tumor microenvironment has a fundamental role in inducing and maintaining drug resistance even in the absence of tumor-specific alterations [reviewed in Ref. (10)]. Tumor microenvironment-associated mechanisms of drug resistance include, but are not limited to, elevated production of growth factors (e.g., HGF) that induce the activation of survival pathways that are not targeted by current therapies [e.g., stimulation of MET receptor by HGF in melanoma cells harboring the mutated form of BRAF(V600E) (11)]; the recruitment of pro-tumorigenic immune cells to the tumor sites elicited by the treatment itself and leading to worse prognosis (12); tumor microenvironment-dependent hypoxia, and high interstitial fluid pressure that may induce cell survival genes and inhibit the distribution of drugs to the tumor site(13). Based on numerous recent studies and on the increased interest in the tumor microenvironment, it has become apparent that targeting not only the cancer cells but also their surroundings may be a novel therapeutic strategy to overcome current limitations. In this regard, it is important to investigate the mechanisms by which the tumor cells can “educate” the surrounding environment to transition from “normal” to pro-inflammatory and pro-tumorigenic. In this scenario, understanding the cross-talk between tumor cells and tumor microenvironment has become of paramount importance. Alongside with other well-known pathways by which cells can communicate (e.g., paracrine and endocrine signaling, adhesion molecules, cell junctions), considerable attention is now being given to the role of extracellular vesicles (EVs) and their protein and nucleic acid cargo. Importantly, EVs allow for the horizontal transfer of information between cells and may account for the existence of tumor-specific genetic alterations in the tumor microenvironment (14).

## Extracellular Vesicles and Their Payload: microRNAs as Intercellular Communicators and Cancer Biomarkers

**Figure 1:**
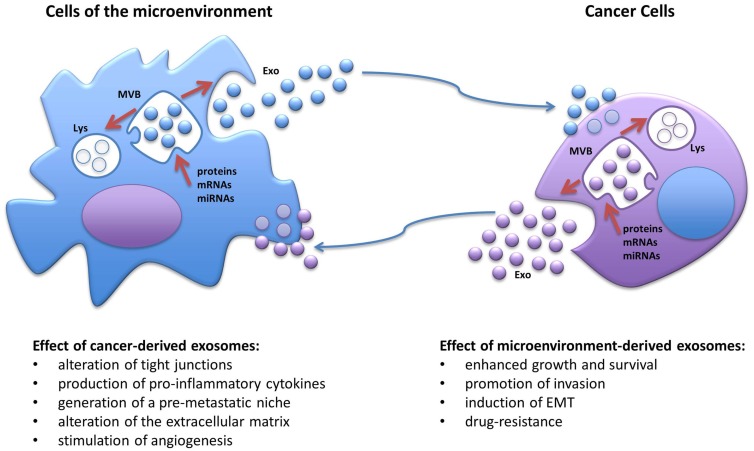
**Schematic representation of the biogenesis of exosomes and the cross-talk between cancer cells and the cells of the tumor microenvironment**. MVB, multivesicular body; Lys, lysosome; EMT, epithelial-to-mesenchymal transitions; Exo, exosomes.

Extracellular vesicles represent a class of circulating cellular fragments, other than apoptotic bodies that can be identified based on their size, generally ranging from 30 nm to a few microns, their density, and the presence of a bilayer lipidic membrane resembling the plasma membrane. The universally recognized categories of EVs are (1) exosomes, with a size of approximately 30–100 nm and a density of 1.13–1.19 g/ml, (2) microvesicles (also known as ectosomes), a more heterogeneous and less characterized group with sizes ranging from 100 to 1000 nm and with a lower density than exosomes, and (3) the more recently discovered “large oncosomes,” derived from bulky cellular protrusions, ranging approximately 1–10 μm in size (15, 16). Although current purification methods do not always allow for the precise separation of larger exosomes and smaller microvesicles due to their sometime overlapping size, their biogenesis is profoundly different (15). While microvesicles originate directly from an outward budding of the plasma membrane and intracellular space, exosomes undergo active packaging in intracellular endosomes, which evolve into multivesicular bodies as a consequence of inward budding of the plasma membrane, and then are either targeted to the lysosomes for degradation or released in the extracellular milieu after fusion with the membrane (Figure 1) (14, 15). Released exosomes can then be targeted to other cells through a very specific yet poorly understood mechanism, likely involving surface proteins, such as tetraspanins and adhesion molecules (15); nonetheless, the exosomes are taken up by the target cells through direct membrane fusion or endocytosis and can be directed to lysosomes for degradation, or their cargo can be released inside the recipient cells (Figure 1) (15). Indeed, all EVs have been shown to contain a specific payload of fully functional proteins and nucleic acids in the form of mRNAs, microRNAs, and long non-coding RNAs (14, 15). In this review, we focus on the role of exosomal microRNAs. MicroRNAs represent a large and ever-growing family of small non-coding RNAs (19–24 nucleotides) that have the ability to deeply control gene expression by recognizing target sequences usually on, but not limited to, the 3′-UTR of specific mRNAs. Partial complementarity of the miRNA sequence to its target will prime the mRNA for degradation or, more frequently, will impede its translation, generally resulting in down-regulation of the encoded protein, although it has been reported that in a few cases, a microRNA may induce higher translation rate of specific mRNAs (14). Since a single microRNA can have multiple targets and a specific mRNA can be targeted by several microRNAs, these small nucleic acids have emerged as fundamental global regulators of gene expression and their levels are frequently altered in cancers and other diseases (14). Importantly, the discovery that exosomes carry a non-random cargo of miRNAs and that they can deliver their content to target cells, raised the fascinating possibility that such ubiquitous nanoparticles could work as a novel category of intercellular communicators both in a paracrine and endocrine fashion (14, 17, 18); the delivery of fully functional miRNAs derived from cancer cells would have the ability of profoundly influence the gene expression of other normal cells both in close proximity, molding the surrounding microenvironment into a cancer-growth permissive milieu, and in distant organs, by possibly transforming a normal site into a pre-metastatic niche. Moreover, since cancer-derived exosomes can be detected in all body fluids, one of the first practical applications in this regard was to exploit their presence as novel non-invasive biomarkers for cancer diagnosis as the expression of specific miRNA families, also known as oncomiRs, is frequently enhanced in several cancers (e.g., miR-155, miR-21, the miR-17–92 cluster, miR-210, miR-16) and their altered expression can be detected in serum from cancer patients when compared to healthy individuals (3, 19). Importantly, changes in the expression of such miRNAs during treatment have also been proposed as a tool for monitoring therapy effectiveness and risk of relapse (3, 19).

Several groups have reported that the loading of proteins and nucleic acids is an active mechanism that requires sorting of specific molecules into the multivesicular bodies where exosomes are being assembled, in a process that is still poorly understood. The current knowledge regarding the loading of proteins through Endosomal Sorting Complexes Required for Transport (ESCRT) and tetraspanin-dependent and -independent mechanisms has been reviewed in Ref. (17). Concerning the loading of microRNA molecules, there is some evidence that the recognition of two different 4-nucleotide motifs by the sumoylated RNA-binding protein hnRNPA2B1 may play a role in the sorting of specific microRNAs into exosomes, and that the availability of microRNAs free to be loaded into exosomes may also be dictated by the abundance of their endogenous RNA targets (i.e., mRNA and other non-coding RNAs) that can therefore act as miRNA “sponges” (17, 18). Interestingly, it has been recently reported that microRNAs can also be found in their precursor state (pre-miRNA) associated to its processing complex (e.g., Dicer, Ago2, and TRBP) inside breast cancer-derived exosomes, where they can be processed into mature microRNAs (20).

## Molding of the Microenvironment by Exosomal MicroRNAs: A Two-Way Communication Highway between Cancer Cells and Their Surroundings

The natural evolution of cancer cells is a multistep process that requires acquisition of growth and survival advantage but also the ability to interfere with the surrounding, shaping the microenvironment into a pro-inflammatory and pro-tumorigenic niche. It is becoming more and more evident that in order to deeply take control over their neighboring normal cells, cancer cells utilize exosomes, which, at least in part, by virtue of their miRNA-cargo, can alter their behavior and induce cancer-promoting functions, such as proliferation, ECM remodeling, migration, invasion, angiogenesis, and metastatic process (Figure 1) (21). It has been shown that exosomal miRNAs can affect cells of the tumor microenvironment both in a canonical (mRNA-targeting) and non-canonical (receptor-binding) manner (6). We have previously reported that NSCLC secretes an abundance of exosomes containing miR-21 and miR-29a and that these particles are released in the microenvironment and taken up by surrounding TAMs and transferred to their endosomes (6). When the exosomal cargo is released, these miRNAs are able to directly bind and activate the endosomal TLR8 receptor (homologous of TLR7 in mice) and induce NF-κB-dependent transcription, this resulting in the expression of the pro-inflammatory cytokines IL-6 and TNF-α that support the growth of NSCLC and its metastatic potential (Table 1) (6). This was the first report of a miRNA to be able to directly activate a receptor. Moreover, we have recently uncovered that a similar mechanism of “education” of TAMs occurs also in the neuroblastoma (NBL) microenvironment and contributes to the development of drug resistance (5). NBL-derived exosomes carry and transfer miR-21 into neighboring monocytes where it activates the TLR8 receptor and induces NF-κB-dependent transcription of miR-155, an oncomiR found frequently overexpressed in cancer (Table 1) (5). Importantly, miR-155 is then shuttled back to the NBL cells packaged in TAM-derived exosomes where it directly targets the mRNA of the inhibitor of telomerase TERF1, resulting in alteration of telomerase activity, telomere length, and overall acquisition of increased resistance to cisplatin (CDDP) treatment. This study raises the intriguing possibility that the frequent increase in expression of miR-155 in solid tumors may be, at least in part, due to the presence of pro-tumorigenic immune cells in the tumor stroma rather than being expressed by the tumor cells themselves. Interestingly, signaling through the murine TLR7 receptor activated by miR-21 delivered by cancer exosomes has also been described to induce cell death of mouse myoblasts (22). This recent observation supports a potential role of exosomes in inducing cancer-associated cachexia, a debilitating muscle wasting syndrome frequently observed in the skeletal muscles of advanced stage cancer patients (22). One of the fundamental characteristics in the evolution of cancer is the ability to invade the microenvironment and give rise to distant metastasis. The epithelial-to-mesenchymal transition (EMT) represents a critical step of tumor progression and malignant transformation, during which cancer cells detach from the primary tumor site, acquire increased motility and the ability to invade the local ECM, followed by extravasation into the bloodstream for dissemination to distant organs (23). The role of intercellular communication for the regulation of EMT and for the induction and maintenance of a pre-metastatic cancer phenotype has been the subject of several studies and the role of exosomal miRNAs has emerged. For example, exosomal miR-223 is transferred from macrophages to breast cancer cells and promotes invasion through down-modulation of the Mef2c/β-catenin pathway (Table 1) (24). Transfer of cancer-derived exosomal miR-105 to endothelial cells has been shown to disrupt the vascular endothelial barrier (i.e., by targeting the tight junction protein ZO-1) during early breast pre-metastatic niche formation (Table 1) (25). Another group has shown that tumor-derived exosomal miR-494 and miR-542-3p were able to modify distant lymph nodes and lung tissue toward a pre-metastatic phenotype suitable for tumor cell hosting, by targeting cdh17 and MAL, and cdh17 and TRAF4, respectively (Table 1) (26). It has recently been shown that an exosome-mediated cross-talk between endothelial cells and breast cancer cells can modulate angiogenesis and tumor growth (27). When HUVEC cells were co-cultured with breast cancer cells (i.e., MDA-MB-231 cells), exosomes derived from endothelial cells showed lower levels of the anti-angiogenic and tumor-suppressive miR-503; while it is not clear how the cancer cells were inducing this change in the endothelial cells, this report highlights a mechanism by which a tumor can silence anti-tumorigenic factors normally secreted by the microenvironment (Table 1) (27). Exosome-associated miRNAs are also involved in the metastatic potential of breast cancer; in fact, it has been shown that metastatic MDA-MB-231 breast cancer cells produce exosomes with higher levels of miR-10b than non-metastatic MCF-7; interestingly, when non-malignant breast epithelial HMLE cells were exposed to exosomal miR-10b, they acquired a more invasive phenotype (Table 1) (28). In a similar fashion, EVs derived from metastatic breast cancer cells were shown to be able to transfer their metastatic potential through delivery of miR-200 to non-metastatic cells (29). These observations are in line with another previous study showing that exosomes can transfer the metastatic activity of highly metastatic BL6–10 melanoma tumor cells to poorly metastatic F1 melanoma tumor cells *in vitro* (30). Exosomal miRNAs have also been shown to be able to induce angiogenesis under hypoxic conditions typical of the bone marrow microenvironment; in fact, multiple myeloma cells grown under chronic hypoxic conditions produce a higher amount of exosomes than cells cultured in normoxic conditions; furthermore, their exosomes had higher levels of miR-135b that, when transferred to surrounding endothelial cells, targets the HIF-1α inhibitor FIH-1 and results in enhanced endothelial tube formation and angiogenesis (Table 1) (31). Several studies have also reported that human mesenchymal/stromal cells (hMSCs) create a tumor-supportive microenvironment, and a recent important report sought to investigate the contribution of their secretome on their pro-tumorigenic potential (32). Specifically, the composition of the cargo of hMSC-derived exosomes was characterized by next generation sequencing and proteomic analysis. The authors found that hMSC exosomes carry a large number variety of tumor-supportive proteins (e.g., PDGFR-b, TIMP-1, and TIMP-2) and of miRNAs (e.g., miR-21 and miR-34a) that exhibited pro-tumorigenic functions when expressed in MCF-7 breast cancer cells (Table 1) (32).

**Table 1:** **Exosomal microRNAs shuttling between cancer cells and tumor microenvironment**.

miRNA	From	To	Target	Effect on TME	Reference
miR-21	NSCLC	TAM	TLR8	⇑Growth and metastasis	(5, 6, 22)
	NBL	TAM		⇑miR-155	
	Lung or pancreatic cancer	Myoblasts		⇑Cachexia	
miR-29a	NSCLC	TAM	TLR8	⇑Growth and metastasis	(6)
miR-155	TAM	NBL	TERF1	⇑Drug resistance	(5)
miR-223	TAM	Breast cancer	Mef2c	⇑Invasion	(24)
miR-105	Breast cancer	Endothelial cells	ZO-1	⇓Tight junctions	(25)
miR-494	Adenocarcinoma	Lymph nodes	MAL; cdh17	⇑Pre-metastatic phenotype	(26)
		Lung	
miR-542-3p	Adenocarcinoma	Lymph nodes	Cdh17; TRAF4	⇑Pre-metastatic phenotype	(26)
		Lung	
miR-503	Endothelial cells	Breast cancer	N/A	⇓Angiogenesis	(27)
miR-10b	Metastatic breast cancer	Non-metastatic breast cancer	HOXD10; KLF4	⇑Metastasis	(28)
miR-200	Metastatic breast cancer	Non-metastatic breast cancer	ZEB1; ZEB2	⇑Metastasis	(29)
miR-135	Multiple myeloma	Endothelial cells	FIH-1	⇑Angiogenesis	(31)
miR-34a	MSC	Breast cancer	N/A	⇑Growth	(32)

*NSCLC, non-small cell lung cancer; NBL, neuroblastoma; TAM, tumor-associated macrophages; MSC, mesenchymal stem/stromal cells*.

## Conclusion

In order to successfully develop advanced therapeutic options for the treatment of cancer, it is becoming increasingly apparent that one cannot disregard the role of the tumor microenvironment. As outlined in this review, the tumor and its surroundings embody a dynamic environment in which cancer cells orchestrate the alteration of exosome-dependent signals that “educate” the microenvironment to act in a pro-tumorigenic fashion. Understanding this cross-talk is of tremendous importance; in fact, one can envision that in the near future, we will be able to counteract pro-tumorigenic and pro-metastatic signals that contribute to the growth, spreading, and drug resistance of tumor cells by potentially engineering the miRNA and protein cargo of exosomes or by interfering with their trafficking.

## Conflict of Interest Statement

The authors declare that the research was conducted in the absence of any commercial or financial relationships that could be construed as a potential conflict of interest.
